# Immobilised Inulinase from *Aspergillus niger* for Fructose Syrup Production: An Optimisation Model

**DOI:** 10.3390/foods13131984

**Published:** 2024-06-24

**Authors:** Marco Lara-Fiallos, Yomira T. Ayala Chamorro, Rosario Espín-Valladares, Juan Carlos DelaVega-Quintero, Valeria Olmedo-Galarza, Jimmy Nuñez-Pérez, José-Manuel Pais-Chanfrau, Amaury Pérez Martínez

**Affiliations:** 1 School of Agroindustry, Universidad Técnica del Norte, Ibarra 100150, Ecuador; 2 School of Agroindustry, Universidad Estatal Amazónica, Puyo 160150, Ecuador

**Keywords:** enzymatic inmobilisation, fructose, inulinase, total productivity

## Abstract

Fructose is a carbohydrate with essential applications in the food industry, mainly due to its high sweetness and low cost. The present investigation focused on optimising fructose production from commercial inulin using the enzymatic immobilisation method and applying the response surface methodology in a 12-run central composite design. The independent variables evaluated were the pH (−) and temperature (°C). The substrate consisted of a commercial inulin solution at a concentration of 1 g/L, while the catalyst consisted of the enzyme inulinase from *Aspergillus niger* (EC 232-802-3), immobilised in 2% *m*/*v* sodium alginate. A stirred vessel reactor was used for 90 min at 120 rpm, and quantification of reducing sugars was determined using DNS colorimetric and UV–Vis spectrophotometric methods at a 540 nm wavelength. After applying the response surface methodology, it was determined that the catalytic activity using the immobilisation method allows for a maximum total productivity of 16.4 mg/h under pH and temperature of 3.9 and 37 °C, respectively, with an efficiency of 96.4%. The immobilised enzymes’ reusability and stability compared to free enzymes were evaluated, obtaining activity up to the fifth reuse cycle and showing significant advantages over the free catalyst.

## 1. Introduction

The generic production of fructose is carried out by the hydrolysis of starch involving enzymes that are fundamental components in different scientific and technological processes; however, the absence of reliable and concrete data on the use of these elements has caused these catalysts to be used in specific areas of research and not in the purely industrial part; at present, biotechnological [1], food [2], clinical [3] and pharmaceutical applications are the most frequent [4]. However, to take advantage of the catalytic activity of enzymes, the conditions of temperature, pH or osmotic pressure must be kept constant, which has limited their use in industrial processes [5]. On the other hand, the solubility of enzymes in the substrate makes their recovery and reuse impossible, which makes the processes costly and unprofitable [6].

Currently, enzyme immobilisation methods represent an alternative to conventional enzymatic processes; these techniques are commonly applied to increase enzyme stability because immobilised enzymes exhibit enhanced operational stability, allowing for repeated use over multiple batch runs without significant loss of activity. This stability is crucial for industrial-scale production processes. It can also be quickly recovered and reused, leading to cost savings and increased efficiency in production processes [7]. Immobilised enzymes can be used in continuous operational modes, such as packed-bed bioreactors, which can lead to higher productivity compared to traditional batch processes, and immobilization can protect enzymes from factors that may cause inactivation, such as pH adjustments or heat treatments, ensuring prolonged enzyme activity during the production process. Immobilization can also allow for better control over reaction conditions, such as substrate concentrations and reaction times, leading to optimised product yields and quality. Immobilised enzymes are often easier to handle and separate from the reaction mixture, simplifying downstream processing and purification steps [8].

On the other hand, the initial setup cost for immobilising enzymes can be higher than for free enzymes, as it may involve additional materials and equipment for the immobilisation process. Immobilisation can sometimes lead to diffusion limitations, where the substrate or product molecules have difficulty accessing the active sites of the enzyme, potentially reducing reaction rates and, depending on the immobilisation method used, there may be a risk of enzyme denaturation or loss of activity during the immobilisation process, which can impact overall enzyme efficiency. Immobilisation methods have limitations in the amount of enzyme that can be loaded onto the support matrix, which could affect the overall catalytic activity of the system. Immobilised enzymes may be more susceptible to substrate inhibition than free enzymes, which could affect the efficiency of the enzymatic reaction. Some immobilisation methods may irreversibly bind the enzyme to the support matrix, limiting the ability to recover and reuse the enzyme for multiple cycles [8].

There are several enzyme immobilisation methods, each of which has different levels of complexity and efficiency during the catalytic process. These methods can be classified into physical retention and chemical binding [9]. Physical methods are based on non-covalent interactions, whereas chemical methods involve establishing covalent bonds between the enzyme and the support [10]. Either way, some variables affect the immobilised enzyme activity because the choice of support material can significantly impact the activity of immobilised enzymes. Different supports have varying affinities for enzymes and can affect enzyme stability and activity. The choice of support material can significantly impact the activity of immobilised enzymes. Different supports have varying affinities for enzymes and can affect enzyme stability and activity. The optimal temperature and pH for enzyme activity may differ between free and immobilised enzymes. The support material and immobilisation process can affect the enzyme’s sensitivity to temperature and pH changes. The type and concentration of cross-linking agents used during immobilisation can affect the stability and activity of the immobilised enzyme. Proper selection and optimisation of cross-linking conditions are crucial. The substrate concentration being acted upon can influence the activity of the immobilised enzyme. High substrate concentrations may lead to substrate inhibition or affect enzyme kinetics [11].

Various matrices have been used for enzyme immobilisation, both of natural or synthetic origin; any material considered for this purpose must present specific characteristics such as mechanical and microbial resistance, thermo-stability, chemical durability, biodegradability, and low cost [12]. Matrices, such as chitosan [13], chitin [14], alginate or silica [15], have been used as enzyme immobilisation media, achieving good results in these processes. In addition, a widely used immobilisation strategy consists of using glutaraldehyde as a support activator to bind to the enzyme covalently, achieving higher enzyme stabilisation [16].

Sodium alginate, whose structure consists of α-L-guluronic (G) and β-D-mannuronic (M) acids, is a polysaccharide from certain brown algae (*Laminaria hyperborea*, *Ascophyllum nodosum* and *Macrocystis pyrifera*) [17], of which 40% of its dry weight is accounted for [18]. Sodium alginate is a versatile biomaterial widely used as a food stabiliser, encapsulation polymer, viscosity enhancer and film former in pharmaceuticals; its use is widespread due to its low toxicity and biodegradability [19]. Sodium alginate is used for immobilisation because is biocompatible, making it suitable for immobilising microorganisms without causing harm. It is biodegradable, ensuring minimal environmental impact and promoting sustainability. It can form gels with calcium ions, providing a solid matrix for trapping microorganisms and contaminants, and immobilised microorganisms in alginate matrices can be recovered and reused, increasing the efficiency and cost-effectiveness of treatment processes [20,21].

Inulinases are enzymes belonging to the family of glycoside hydrolases, also known as glucosidase or debranching enzymes, that catalyse the hydrolysis of inulin to fructose; these enzymes are capable of hydrolysing inulin into practically pure fructose and, in smaller proportions, hydrolysing it into glucose [22]. Inulin is a water-soluble storage polysaccharide that belongs to a group of non-digestible carbohydrates called fructans. It is considered a dietary fibre and is commonly found in over 36,000 species of plants, with chicory roots being one of the richest sources of inulin. Inulin has attained Generally Recognised as Safe (GRAS) status in the USA and is widely used in various food products for its functional properties and health benefits. It is resistant to digestion in the small intestine but can be fermented in the large intestine, serving as a substrate for beneficial gut bacteria. Inulin has a low caloric value and is known for its prebiotic effects, aiding in digestive health and potentially offering other health benefits, such as improved lipid metabolism [23].

The chemical structure of inulin consists of linear (2 → 1)-linked β-D-fructosyl units attached to the fructosyl moiety of sucrose. The number of fructose units in inulin can vary, typically ranging from 2 to 60 in chicory inulin, indicating a combination of oligomers and polymers. The degree of polymerisation (DP) and branching of inulin molecules can affect their functionality. Plant-derived inulin generally has a lower DP (maximally < 200) compared to bacterial inulin, which can have a very high DP, ranging from 10,000 to above 100,000, and is more branched than plant-derived inulin [24]. Various physico-chemical characteristics influence the behaviour of inulin, such as its degree of polymerisation (DP), size distribution, molecular weight, flexibility, and interactions with solvents. Inulin can form gels under certain conditions, creating networks through micro-crystallisation and solid–solid interactions. Inulin’s flexible backbone and high glass transition temperature (Tg) make it an ideal stabiliser for proteins in dry states for food and pharmaceutical uses [25].

Regarding rheological behaviour, inulin solutions exhibit viscosity and gelling properties, which factors such as concentration, temperature, and shear stress can influence. The storage stability of proteins during freeze-drying is influenced by the size and flexibility of inulin molecules. Additionally, inulin can be used to form hydrogels for drug delivery applications through UV-photo crosslinking of inulin derivatives [25].

Fructose research is interesting because of its potential impact on human health and product development. Fructose aids in storing energy and regulating blood sugar in the liver. Some studies suggest that fructose affects satiety and hunger hormones differently than glucose, which could impact appetite regulation. Manufacturers can use it as a sugar composition for beverages instead of high-fructose corn syrup, which researchers link to obesity, metabolic diseases, adverse effects on cardiovascular health, and effects on appetite regulation, which lead to overeating. Fructose can be used to produce new products, like butyl levulinate, which companies can then use as a blending oxygenate additive for gasoline and diesel; this can lead to improved fuel quality and reduced pollutants [26,27,28].

Enzymatic hydrolysis by inulinases has been considered the most promising technique for obtaining fructose syrup from inulin because it achieves yields of up to 95% [29]. Based on these aspects, the objective of this work focuses on the study of the most suitable conditions for the hydrolysis reaction of inulin using inulinase encapsulated in sodium alginate matrices to produce fructose syrup; the primary experimentation variables were pH and temperature because they are considered the fundamental parameters for the activation of the catalyst. There are no reports that this mechanism has been used under the specific conditions employed in the experiment for generating fructose syrup, nor have the process conditions been optimised; this can be applied to unused organic wastes that can be worked on a large scale based on consumption of daily life, such as peels, rotting vegetables and fruits with inulin content.

## 2. Materials and Methods

Inulinase from *Aspergillus niger* (EC 232-802-3) commercial Sigma-Aldrich (9025-67-6) in aqueous glycerol solution presentation and commercial chicory inulin in white powder presentation (EC 232-684-3; CAS Number: 9005-805-5, Sigma-Aldrich, New York, NY, USA) was used. The specific activity of the enzyme is greater or equal to 200 INU/g, and its density varies between 1.10 and 1.30 g/mL. The specified fructose to glucose ratio was greater or equal to 20:1 with glucose and free fructose percentages of less than 0.05%.

For matrix preparation, 1 g of sodium alginate (GRINDSTED^®^ Alginate FD/PH 120, New York, NY, USA) diluted in 50 of distilled water at 35 °C was used and homogenised vigorously until complete dissolution, obtaining a final concentration of 2% (*m*/*v*) [30]. The solution was allowed to stand at 5 °C for 30 min. After this time, using 13 mL of the sodium alginate solution, 52.5 enzyme activity units (EAUs) corresponding to 100 µL of enzyme diluted 1:1 with distilled water were immobilised. Three mL of the alginate–enzyme mixture was dripped into a 100 mL vessel containing 60 mL of 10% CaCl_2_ solution, and the beads formed were allowed to stand in the calcium solution for 30 min at 5 °C. Finally, the capsules (5 mm of diameter) were recovered by filtration and washed with a good amount of distilled water to remove calcium chloride residues. The efficiency of immobilisation was analysed through the diffusivity and the time the alginate spheres can be kept with a firm consistency, which helped to determine the number of enzymatic units present in the bead and verify its stability.

The catalytic activity was evaluated considering the theoretical optimal pH (5) and temperature (50 °C) values of inulinase from *Aspergillus niger* [31] and values studied in previous investigations, in which sodium alginate was used as an immobilising agent [32]. Each experimental unit consisted of 25 mL of 1% (*m*/*v*) substrate (Inulin from chicory^®^, Sigma-Aldrich, New York, NY, USA) and 12.5 mL of enzyme solution gelled in 10% calcium chloride. The batch reaction was carried out in a QBT-200/400D MRC digital bath with automatic temperature control and constant agitation of 120 rpm, and the pH was regulated every 30 min with 0.05 M citric acid. At the end of the process, the samples were subjected to heat shock with ice water at 4 °C to slow the reaction, and the encapsulated enzymes were separated from the final product by filtration. The experiment involved hydrolysing 50 mL of a 1% (*m*/*v*) commercial inulin solution for 180 min at 50 °C and pH 5, using 105 EAU. A sample was taken every 30 min, and fructose was quantified using UV–Vis spectrophotometry at a wavelength of 540 nm; results were replicated three times and were expressed in g/L.

### 2.1. Fructose Quantification

The quantification of reducing sugars was performed by applying the 3.5 dinitro salicylic acid (DNS) method [33]. The DNS reagent was prepared using 2.5 g of 3.5 dinitro salicylic acids, 75 g of sodium potassium tartrate tetrahydrate and 4 g of sodium hydroxide. The sodium hydroxide was dissolved in 100 mL of distilled water with constant agitation, and the sodium-potassium tartrate was added gradually. It was gauged with distilled water up to 200 mL and then 3.5 dinitro salicylic acid was added. Finally, it was gauged up to 250 mL and left in agitation until complete dissolution of the solutes. The reaction consisted of taking samples of 0.5 mL of fructose syrup and placing them in 10 mL glass test tubes, adding 0.5 mL of DNS reagent. The test tubes were subjected to a boiling water bath for 5 min. They were allowed to cool, and 5 mL of distilled water was added. Finally, they were shaken, and readings were taken at 540 nm by UV–Vis spectrophotometry, using a standard curve of standard fructose at different concentrations. The linearity of the quantification curve achieves a R^2^ of 0.9982, with a limit of quantification of 1.027, limit of detection of 0.001 g/L and an uncertainty of 0.28.

### 2.2. Fructose Production Optimization

Response surface methodology (RSM) was applied to optimise fructose productivity after 90 min of reaction using inulinase immobilised on sodium alginate. The use of the central composite design in this experimentation allowed for the obtaining of information on the interaction of pH and temperature in the inulinase activation process. A second-order with interactions model to adjust experimental values was used, and the model quality was tested with ANOVA, verifying the model terms’ validity. Residuals were analysed after verification of normality and randomness of mean central value.

Validation of the optimisation model in the hydrolysis reaction was achieved by detecting the absence of non-linearities in the system between the independent variables and the response, which affects the efficient determination of the system response to changes in multiple selected independent variables, providing robustness of the model throughout the experimental region of interest. A central composite design (CCD) was used, with three levels of each treatment and four center-point replicates (Table 1 and Table 2). The range of independent variables, temperature (*X*1) and pH (*X*2) was adopted from preliminary research [31,34,35,36]. The experiments were planned and analysed using Design-Expert 11.0.3.0 software (Stat-Ease, Inc., Minneapolis, MN, USA).

This experimental approach allowed the identification of optimal conditions to maximise fructose production while ensuring process efficiency and stability. The use of response surface methodology and central composite design facilitated the exploration of the system response to different experimental conditions and the validation of the optimisation model, which significantly contributes to the advancement of efficient fructose production from inulin by immobilising inulinase on sodium alginate spheres.

The response variable was fitted to a second-order statistical model described in the following equation:

(1) PR=f(pH,T)

where *PR* is the total fructose productivity (mg/h) calculated by the Formulas (2) and (3), a dependent function of solution pH and working temperature.

(2) $$PR=\frac{fructose\;concentration\;\left[\frac{mg}{mL}\right]\cdot Solution\;volume\;[mL]}{time\;[h]}$$

(3) $$PR=\frac{produced\;fructose\;[mg]}{time\;[h]}$$

The predicted optimum values for a combination of independent variables were confirmed by performing additional experiments under the same conditions to verify the validity of the statistical model obtained. Once the best treatment was identified, its capacity to reuse the enzymes was verified. This capacity was determined by analysing the hydrolysis of a 1% solution of commercial inulin. A volume of 25 mL of this substrate was subjected to hydrolysis with immobilised enzymes in sodium alginate for 90 min at 37 °C and pH of 4 with constant agitation at 120 rpm. After this period, the enzymes were recovered by filtration and a new volume of unhydrolysed substrate was added to start a new cycle. Enzyme activity was measured by calculating the *PR* in each cycle, and repeats were run until a reading, like the residual fructose content of the commercial inulin blank, was obtained.

Three trials were carried out for free and immobilised enzymes to compare the catalytic capacity. Twenty-five mL of 1% commercial inulin solution were hydrolysed at the optimum conditions obtained after the development of the experimental design, and the time and agitation parameters were kept constant. The enzyme activity was measured by calculating the *PR* after quantification of reducing sugars by UV–Vis spectrophotometry.

## 3. Results

The optimisation of the immobilisation process led to the study of the influence of temperature and pH on total fructose productivity. Table 3 shows the results obtained for each experiment. It can be deduced that the best results were obtained at the high levels for each factor, since fructose productivity had an average reduction of 56% in the trials, including the low levels of the experimental design. This model achieves a standard error rate of 0.44 and a coefficient of variation of 3.51%.

**Figure 1:**
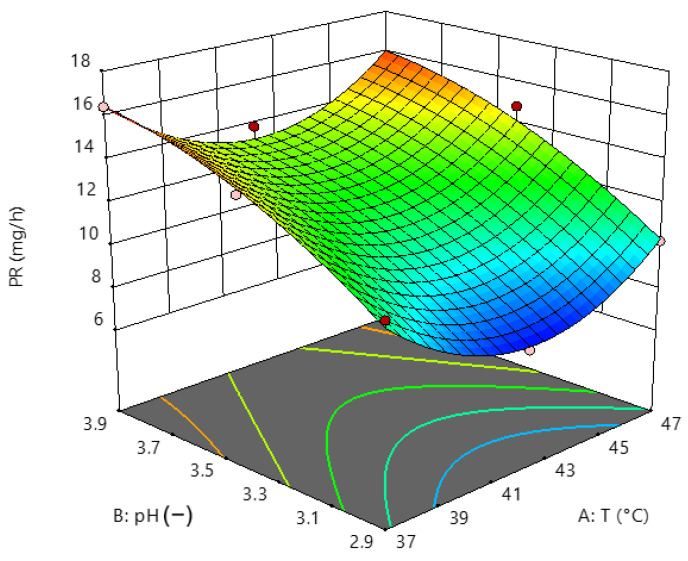
The response surface with the combined effect of temperature (°C) and pH on total fructose productivity (mg/h). Dots are the experimental data, colours represent high (red) to lower (blue) productivity value.

Figure 1 shows the effects of temperature and pH on the dependent variable PR. The zones of higher productivity are shown in red, the middle zone in green, and the lowest values in blue, which translate into unsuitable conditions for the reaction.

**Figure 2:**
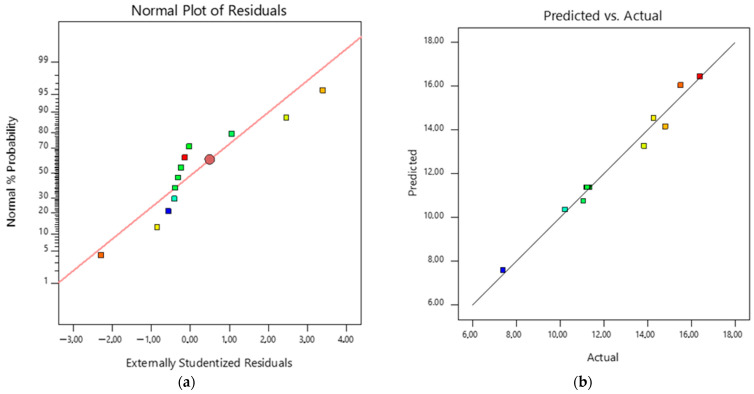
Diagnostic plots: (**a**) standard probability plots of externally studied residues; (**b**) predicted versus actual values of PR (mg/h). Point’s colours are the same from Figure 1.

The diagnostic graphs of the normal distribution of the residuals (Figure 2a) show that the data present normal behaviour since they are randomly located around the central line (R^2^ = 0.982); the graph of the predicted response values versus the actual response values (Figure 2b) made it possible to evaluate the adequacy of the quadratic model for the determination of productivity as a function of the independent variables (temperature and pH); the trend shows that there are no additional variables to be considered in the model. Table 4 shows the results of the statistical analysis for the modelling of the system under study.

Table 4 shows that the *p*-value was less than 0.05 for the pH variable but not for the T variable; this indicates that the factor influencing the process is pH. This agrees with Zittan Bagsvaerd, cited in [37], who, in his experiments, found that the productivity of hydrolysis, seen as the sum of glucose and fructose as a function of total carbohydrate content, is constant at temperatures like those utilised in this research. However, in the same research, it is evident that pH has a considerable influence on the activity of inulin, minimising its effect at values above 5. The present study also shows that the interaction of temperature and pH effectively influences fructose productivity. Thus, the mathematical analysis generated the primary coefficients of the model, and Table 5 shows a standard error of the statistical iteration with low values, showing a good assertiveness of the calculation in the confidence intervals exposed at 95%.

The model for coded values obtained is presented in Formula (4).

(4) $$PR=11.38-0.21\cdot X1+2.84\cdot X2+2.98\cdot {X1}^{2}-0.95\cdot {X2}^{2}$$

*X*1 represents the coded variable for temperature, and *X*2 symbolises the coded variable for pH. With a regression coefficient of 0.982, the model in terms of absolute values is presented in Formula (5).

(5) $$PR=159.76-10.04\cdot T+31.51\cdot pH+0.12\cdot T^{2}-3.8\cdot {pH}^{2}$$

### 3.1. Batch Hydrolysis Experiments

**Figure 3:**
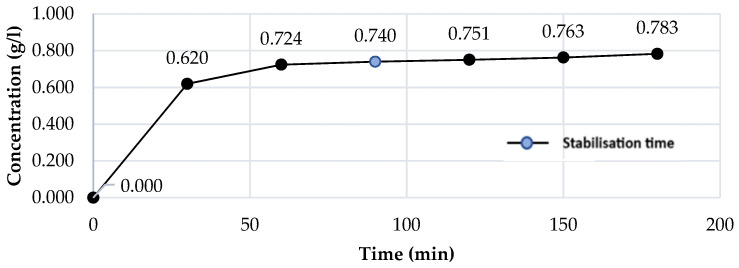
Enzymatic conversion curve of inulin using optimal values of activation of *A. niger* inulinase (T = 37 °C, pH = 3.9).

The results show that, during the reaction, the substrate was converted partially, very close to the totality. Figure 3 shows the behaviour of the enzymatic hydrolysis in the reaction process.

It can be evidenced that the enzymatic conversion process generates a stabilisation regime after 90 min, which is the time in which the process reaches a fructose concentration value of 0.740 (g/L); after this interval, the trend’s growth is not significant for productive purposes due to the energy requirement incurred. The analysis of the inulin conversion kinetics (Figure 3) indicated that the experimental data closely align with both first-order (K_1_ = 0.0013 min^−1^, C_0_ = 0.6369 g/L, R^2^ = 0.7414) and second-order (K_2_ = 0.0018 L·g^−1^·min^−1^, C_0_ = 0.6375 g/L, R^2^ = 0.7165) models. Even though the literature shows that this type of reaction has a first-order kinetic [38], the results of this study do not show a good fit; this may be because different reactions are generated simultaneously in the bead, which does not allow a clean relationship in the data. It is also possible that chemical kinetics do not govern the process, rather the mass transfer through the diffusion of inulin and the effusion of fructose, given that this system is immobilised.

### 3.2. Enzymes’ Reuse

**Figure 4:**
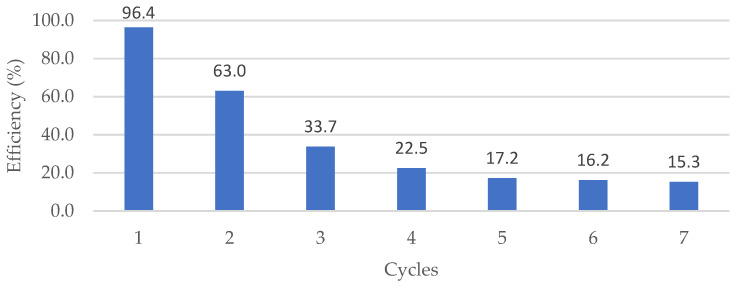
Efficiency curve of *Aspergillus niger* inulinase enzyme immobilised in sodium alginate at the optimum pH (3.9) and temperature (37 °C) of the reaction medium.

The reuse of the immobilised biocatalysts was studied due to the importance of recovering the enzyme for subsequent applications. Figure 4 shows the behaviour of the catalyst in each reuse cycle.

The reuse of inulinase immobilised in sodium alginate was evaluated during seven consecutive cycles for the hydrolysis of 1% inulin at 37 °C for 90 min, a time in which the enzymatic activity is evidenced until the sixth cycle, obtaining a productivity of 2.70 mg/h. The comparison of the productivity based on the reading of the commercial inulin blank quantifies a residual concentration of reducing sugars of 0.155 g/L of fructose, equivalent to a productivity of 2.58 mg/h. In the first cycle, a productivity of 16.07 mg/h of reducing sugars was obtained; however, after this, there was an evident decrease in the conversion; this is due to the dissipation of enzymes bound to the support material after the washing of the beads at the end of each cycle and by the permanent agitation during the process (Figure 4). There are few reports on reusing inulinase immobilised in the batch reactor. According to Rocha et al. [38], the reuse of immobilised inulinase is useful for up to seven cycles for inulin hydrolysis; this fluctuation may be due to several factors, such as support material used, reaction conditions, or the type of process. Other investigations have shown that immobilised inulinase can be reused several times while maintaining the yield of the reaction, as in the case of Catana et al. [39], who evaluated the stability and reusability of Amberlite-immobilised inulinase for inulin hydrolysis. The study demonstrated that the immobilised biocatalyst could be successfully reused in several batch runs (18) at different temperatures without significantly decreasing product yield. This reusability highlighted the operational and mechanical stability of the immobilisation matrix, making it a promising candidate for the efficient production of high fructose syrups from inulinase. The findings suggest that immobilisation enhances the stability and half-life of the enzyme, providing insights into the potential applications of immobilised enzymes in biocatalytic processes. Holyavka et al. [40] focused on efficient fructose production from plant extracts using immobilised inulinases from *Kluyveromyces marxianus* and *Helianthus tuberosus*. The study demonstrated the stability and productivity of the immobilised inulinases through 10 batch runs, each lasting 20 min. The results indicated that the activities of the yeast and plant inulinases remained almost unchanged after these repeated uses, suggesting their potential for industrial applications. Specifically, the yeast inulinase immobilised on AV-17-2P showed high productivity with various plant extracts, making it a promising candidate for fructose production. The research highlights the potential of immobilised inulinases for sustainable and efficient fructose production from plant sources.

### 3.3. Immobilised vs. Free Enzymes

**Figure 5:**
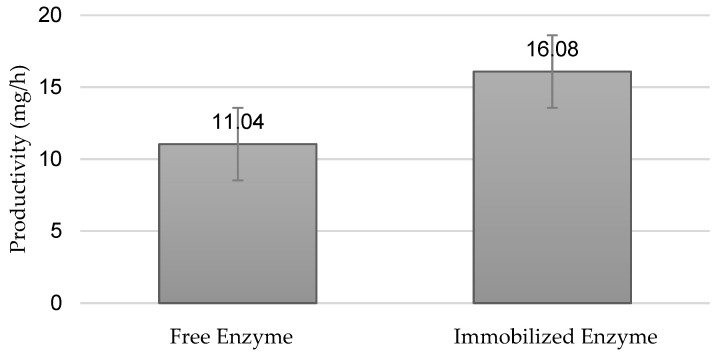
Productivities were obtained using free and immobilised enzymes at the optimum pH (3.9) and temperature (37 °C) of the reaction medium.

Figure 5 shows the results obtained after performing an assay with free and immobilised enzymes. The same reaction conditions were used for this experiment (T = 37 °C and pH 3.9).

It is evident that, at 37 °C, the activity of immobilised inulinase is higher than that of free inulinase, showing that immobilisation positively affected the temperature tolerance of the enzyme during the reaction. This result may be due to the protection exerted by the support material towards the catalyst, preventing heat damage; this achieved higher thermal stability than the free enzyme. Improving stability by immobilising the enzyme could significantly reduce the cost of operation, as it can be reused for extended cycles in continuous or batch processes, since the heterogeneity of the enzyme systems allows easy recovery of both the enzyme and the product [41]. The results obtained in the present work coincide with those of the study by [42], where it is mentioned that, indeed, the enzyme immobilisation method increases the thermal stability of inulinase; likewise, it has been stated that immobilised enzymes are more resistant to high temperatures.

Regarding the parameters studied, optimal temperatures for inulinase activity of *Aspergillus niger* have been reported to be around 50 °C [36], while others reported higher values, reaching 60 °C [35]. Moreover, the optimum pH of inulinase activation in other reports indicates values of 4.3 to 4.5 [31,34]. The results have a close relationship with those obtained in the present investigation. Separating and reusing immobilised enzymes is one of the main advantages of enzyme immobilisation. The data shown in Figure 4 showed that inulinase immobilised in sodium alginate retained 65% of its activity in the second cycle of use while, in the third cycle, 35% of enzyme activity was retained. Enzyme activity was evidenced up to the fifth reuse period, as evidenced by previous research that maintained the activity of enzymes immobilised in sodium alginate for several cycles [43]. The reaction yield reached in previous research is 82% [44], while in the present work a yield of 92.4% was reached, generating a substantial improvement in the process recorded above.

## 4. Conclusions

The response surface method allowed development of a model that faithfully represents the hydrolysis of inulin for fructose generation (R^2^ = 0.982), finding that the optimum productivity point is at 37 °C and 3.9 pH with a yield of 82%, which corresponds to the reaction’s kinetics. The kinetics of the conversion of inulin to fructose syrup shows a favourable data fit; however, there is a possible diffusivity and effusivity influence of inulin and fructose, respectively. There is also an influence of the catalyst effusivity, which has an impact on the kinetics, so it does not completely fit a first-order reaction. With the above conditions, it was evidenced that the enzymes can be reused up to six times. Enzyme immobilisation in sodium alginate for inulin hydrolysis proved to be a technique that enables substrate conversion with significant advantages for industrial applications due to the practicality of the process, reuse, and stability of the catalyst. Advantages are not evident in processes that employ free enzymes suspended directly in the substrate. This work can contribute to establishing a catalytic process that allows mass production of fructose, not only from commercial inulin but also from fructans obtained from other natural sources that contain them, representing a profitable and environmentally friendly process with potential uses in Ecuador’s pharmaceutical and food industry. The experiments show that inulinase can be reused for up to six cycles with good productivity levels, which influence the possibility of using this technology on a large scale.

## Figures and Tables

**Table 1:** Actual and coded independent variables values.

Coded	Temperature (°C)	pH
−1	37	2.9
0	42	3.4
1	47	3.9

**Table 2:** Central composite design runs distribution.

Run	*X*1 ^1^	*X*2 ^2^
R1	0	0
R2	0	−1
R3	1	0
R4	0	0
R5	−1	0
R6	0	0
R7	1	−1
R8	1	1
R9	0	0
R10	−1	1
R11	−1	−1
R12	0	1

^1^ Code for Temperature. ^2^ Code for pH.

**Table 3:** Actual total fructose productivity (PR) values (mg/h) obtained after the trials proposed by the experimental design were developed. Model total fructose productivity and residual.

Run	Actual PR (mg/h)	Model PR (mg/h)
**R1**	**11.28 ± 0.06 ^1^**	11.38 ± 0.44
R2	7.40	7.59 ± 0.44
R3	14.81	14.15 ± 0.44
**R4**	**11.28 ± 0.06 ^1^**	11.38 ± 0.44
R5	14.28	14.57 ± 0.44
**R6**	**11.28 ± 0.06 ^1^**	11.38 ± 0.44
R7	10.23	10.36 ± 0.44
R8	15.52	16.04 ± 0.44
**R9**	**11.28 ± 0.06 ^1^**	11.38 ± 0.44
R10	16.40	16.46 ± 0.44
R11	11.07	10.78 ± 0.44
R12	13.85	13.27 ± 0.44
% CV	3.51	3.51

^1^ Center point replicates. MEAN = 11.28 ± 0.44 mg/h.

**Table 4:** Second-order model analysis of variance (ANOVA) related to fructose productivity as a function of temperature and pH.

Source	Square Sum	DF ^1^	Mean Square	F Value	*p* Value	Significance
Model	72.40	4	18.100	95.43	<0.0001	Significative
A	0.233	1	0.233	1.23	0.3039	
B	48.550	1	48.550	255.97	<0.0001	
A^2^	23.610	1	23.610	124.51	<0.0001	
B^2^	2.400	1	2.400	12.67	0.0092	
Residual	1.330	7	0.189			
Adjustment failure	1.320	4	0.328	79.50	0.0022	Significative
Error	0.012	3	0.0041			
Total	73.720	11				

^1^ Degree of freedom.

**Table 5:** Second-order model coefficients related to fructose productivity as a function of temperature and pH.

Factor	Coefficient	DF ^1^	Standard Error	Low 95% CI ^2^	High 95% CI ^2^	VIF ^3^
Intersection	11.38	1	0.1988	10.91	11.85	
A	−0.20	1	0.1778	−0.62	0.22	1.00
B	2.84	1	0.1778	2.42	3.26	1.00
A^2^	2.98	1	0.2667	2.35	3.61	1.13
B^2^	−0.95	1	0.2667	−1.58	−0.32	1.13

^1^ Degree of freedom. ^2^ Confidence interval. ^3^ Variance inflation factor.

## Data Availability

Data available on request due to privacy or ethical restrictions. The data presented in this study are available on request from the corresponding author.
